# A Case of Supernumerary Kidney

**DOI:** 10.7759/cureus.3686

**Published:** 2018-12-05

**Authors:** Marco Mejia, Joseph Limback, Ashley Ramirez, Jeremy R Burt

**Affiliations:** 1 Radiology, University of Central Florida College of Medicine, Orlando, USA; 2 Radiology, Florida Hospital, Orlando, USA

**Keywords:** supernumerary kidney, congenital kidney, crossed fused ectopia

## Abstract

A supernumerary kidney is extremely rare, with less than 100 cases currently reported in the literature. When this variant is present, the additional renal parenchyma demonstrates its own collecting system, vascular supply, and distinct encapsulated parenchyma. Herein, we discuss the case of a supernumerary kidney in a 20-year-old male.

## Introduction

A supernumerary kidney is one of the rarest congenital anomalies of the urogenital system. There are currently less than 100 cases currently reported in the literature, with the first case being described in 1965 [1]. This anatomic variation is said to occur whenever there are more than two kidneys present, with the additional kidney having its own collecting system, vascular supply, and distinct encapsulated parenchyma. We report a rare case of a fused supernumerary kidney in a 20-year-old male patient.

## Case presentation

**Figure 1:**
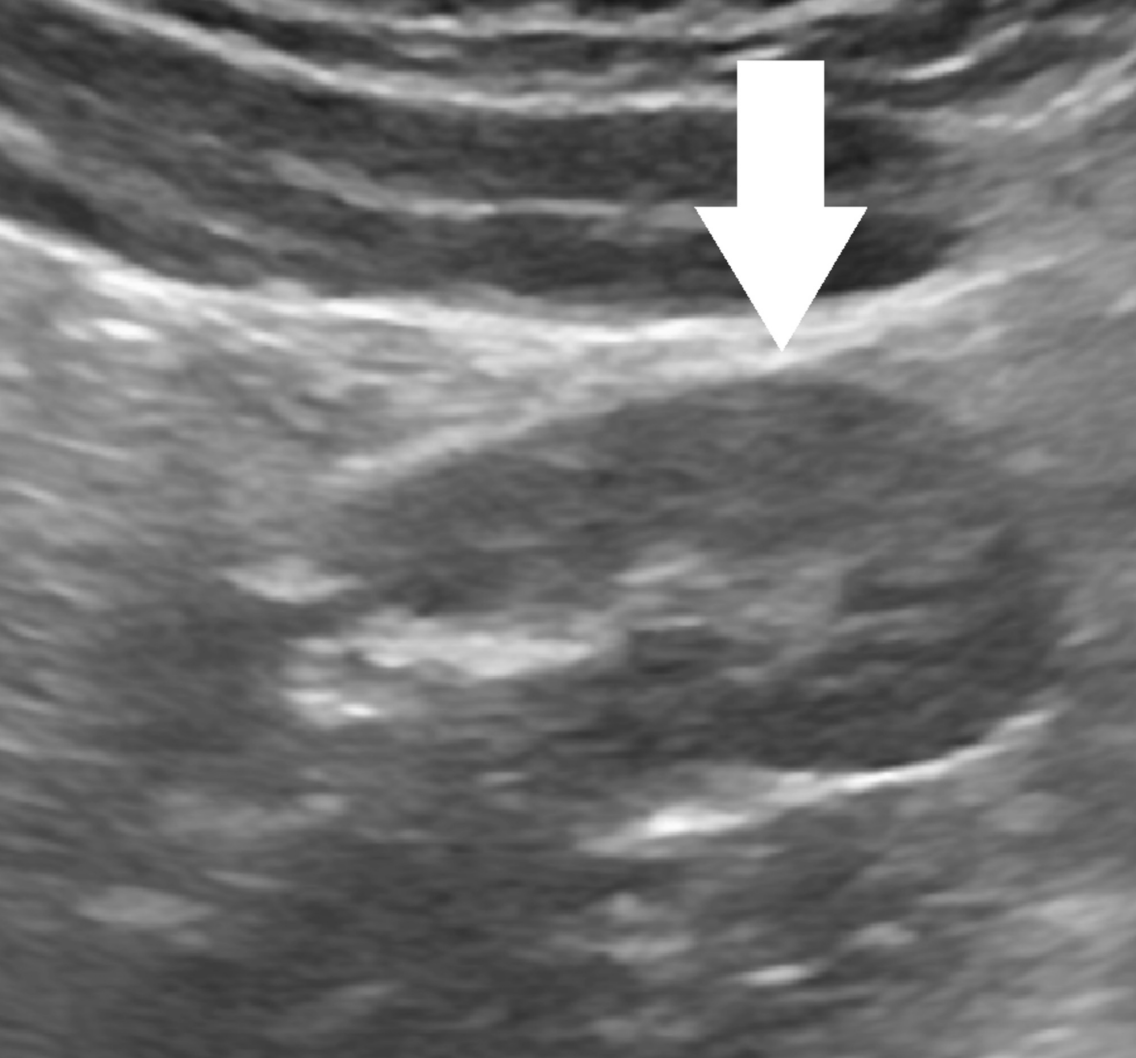
Grayscale Ultrasound Image Grayscale ultrasound image demonstrating a centrally hyperechoic and peripherally hypoechoic right lower quadrant mass without signs of inflammation.

**Figure 2:**
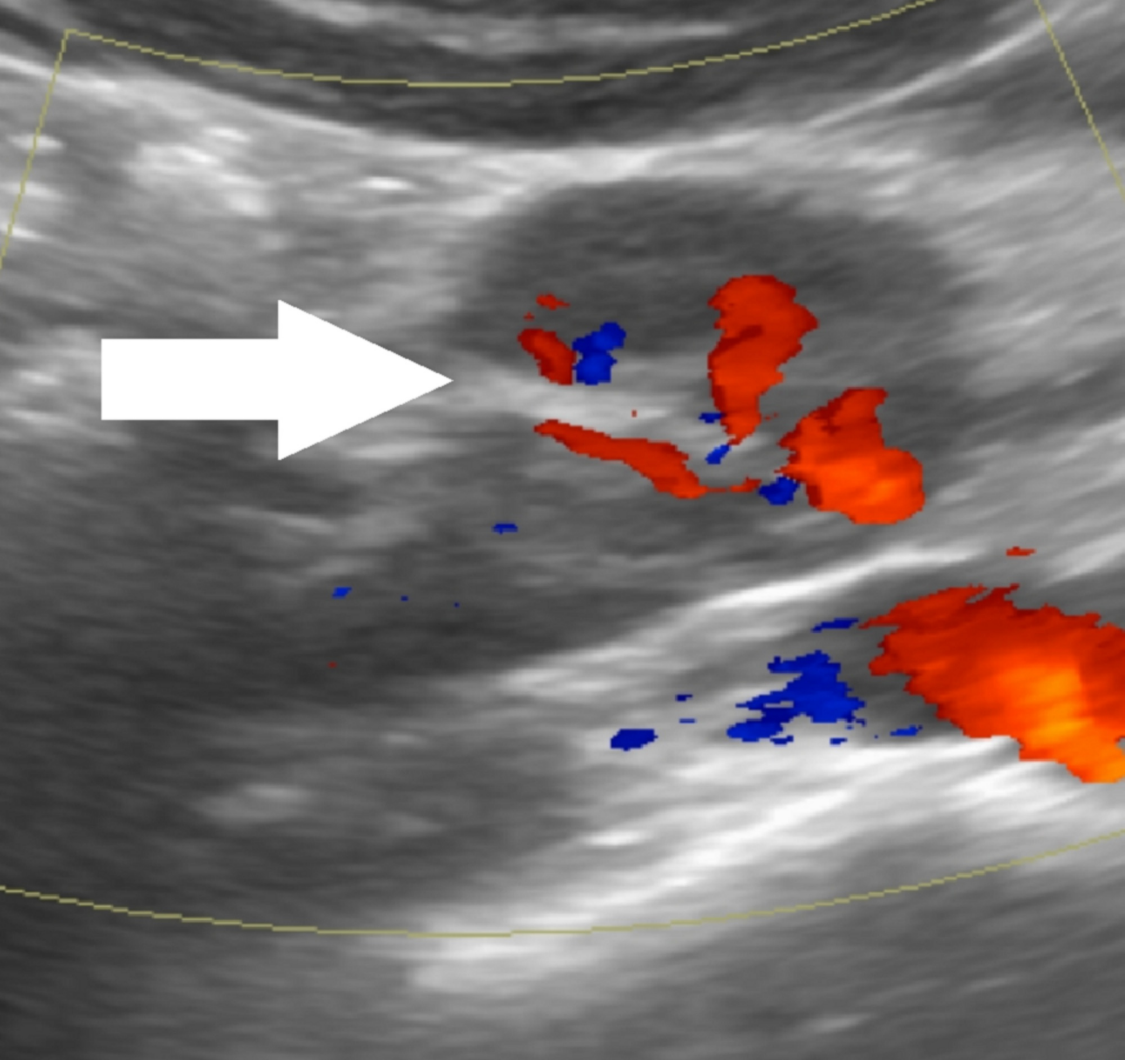
Doppler Ultrasound Image Demonstrating a Vascular Pedicle Doppler ultrasound image demonstrating the reniform appearance of the mass with a central vascular pedicle.

A 20-year-old male with a past medical history of seizures since the age of 14 and treated with divalproex sodium and topiramate presented to the emergency department with right lower quadrant abdominal pain. On presentation, all vital signs appeared normal. A complete metabolic panel and blood count were within normal limits (creatinine of 0.89 mg/dL and blood urea nitrogen of 14 mg/dL). An abdominal ultrasound was performed that demonstrated a nonspecific structure in the right lower abdomen with a vascular fatty central parenchyma, as well as an avascular hypoechoic peripheral parenchyma (Figures 1, 2).

**Figure 3:**
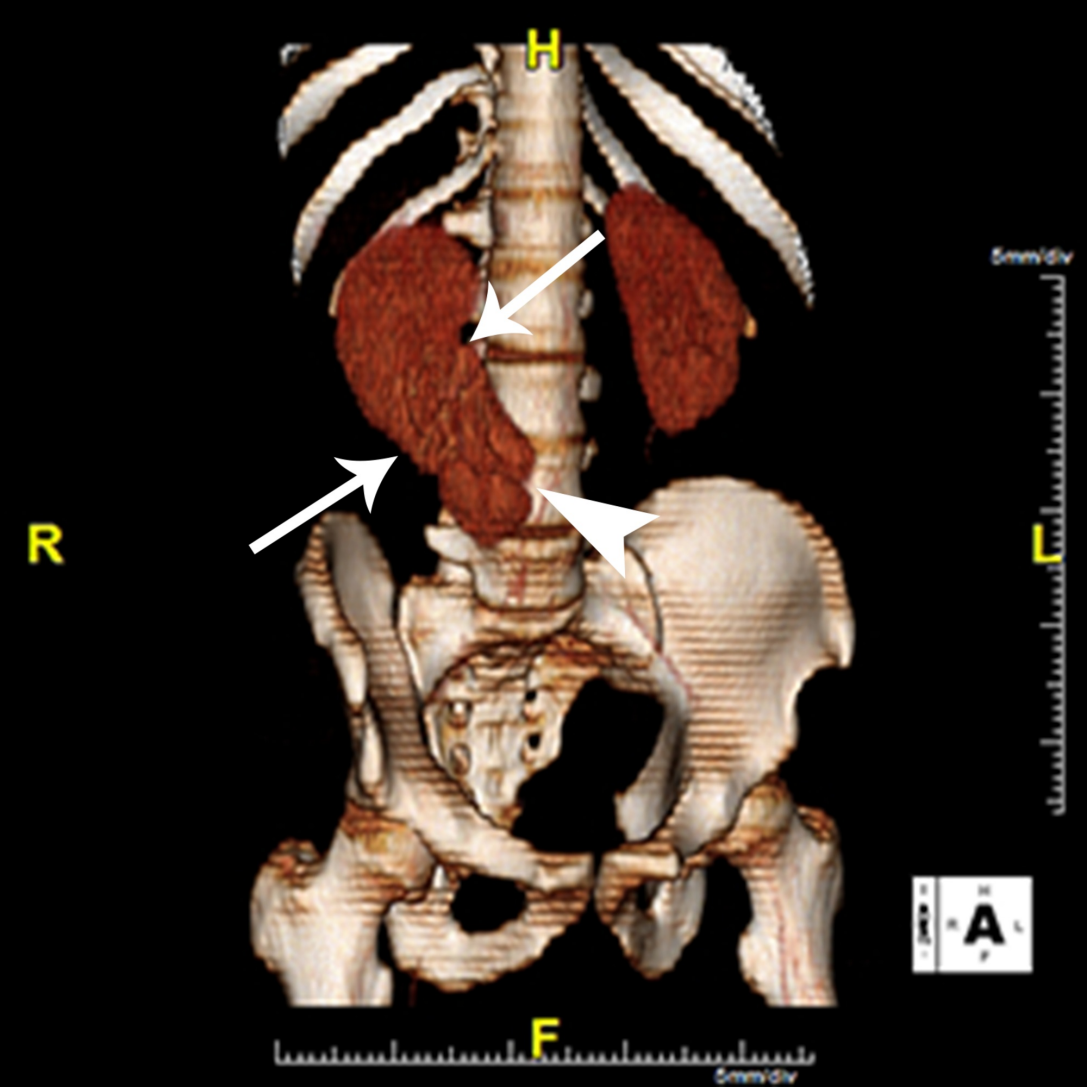
3D Computed Tomography (CT) Reconstruction Three-dimensional (3D) reconstructed CT image of the kidneys demonstrates bilateral native kidneys, as well as an ectopic supernumerary kidney fused to the lower pole of the native right kidney (white arrows). The image also demonstrates the supernumerary kidney hilum (white arrowhead).

**Figure 4:**
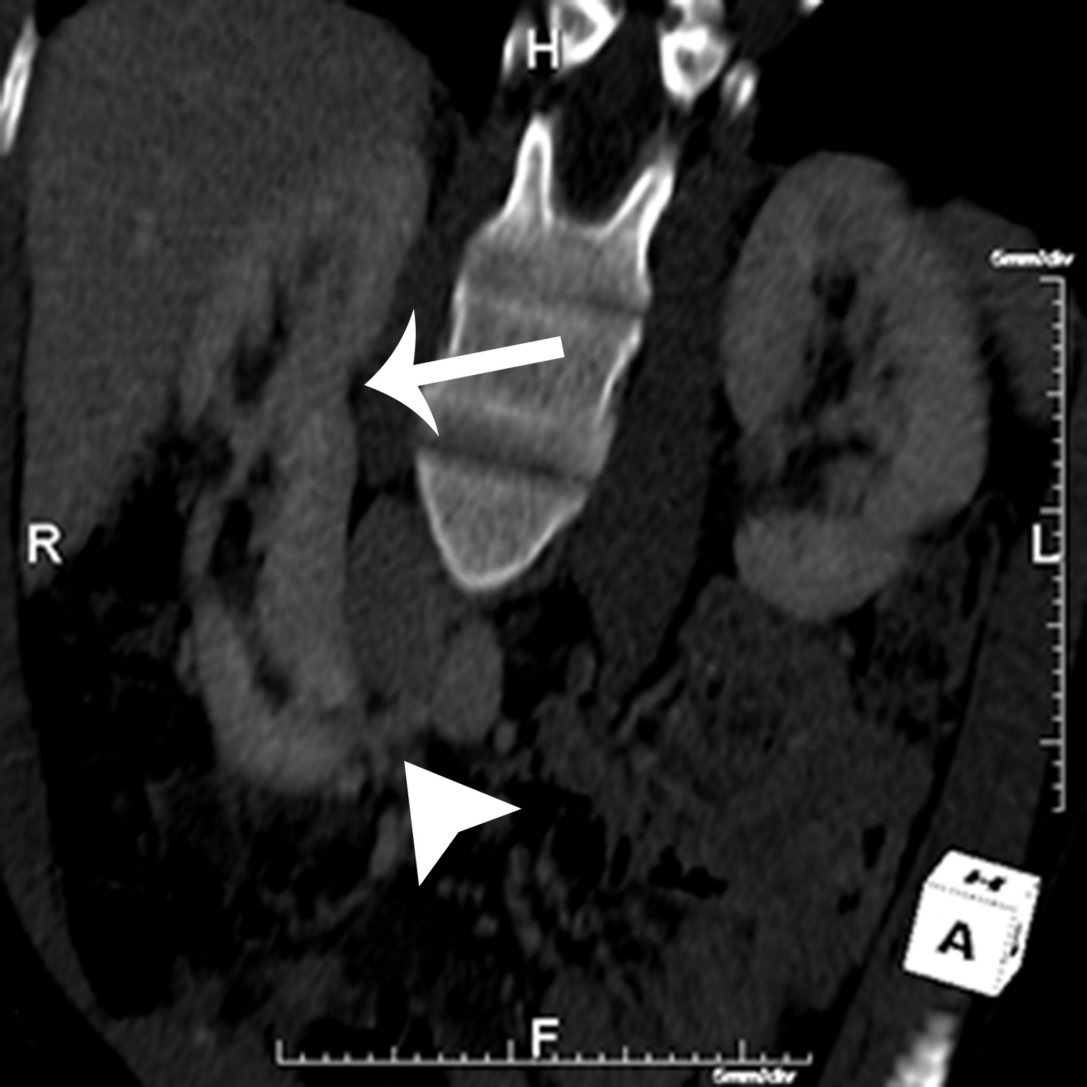
Coronal Oblique CT Reconstruction Coronal oblique reconstructed computed tomography (CT) image of the kidneys demonstrates bilateral native kidneys, as well as an ectopic supernumerary kidney fused to the lower pole of the native right kidney (white arrows). Additionally, the supernumerary kidney renal vein can be seen draining into the inferior vena cava (white arrowhead).

There were no acute findings on ultrasound. A computed tomography (CT) scan demonstrated no acute abnormality, but a supernumerary kidney fused to the lower pole of the native right kidney was visualized (Figures 3, 4).

## Discussion

The supernumerary kidney is an extremely rare congenital anomaly, with less than 100 cases being reported in the literature [1]. This condition is asymptomatic in most cases but, when symptomatic, it most often presents with symptoms in the fourth decade of life. The most commonly reported symptoms are pain, a palpable abdominal mass, and fever [2-3]. Some cases may also present with urinary symptoms, such as urinary incontinence [4]. The diagnosis is made with imaging, which may include computed tomography (CT), magnetic resonance imaging (MRI), or ultrasonography.

Most cases of supernumerary kidney present with one additional kidney. The additional kidney most commonly occurs ipsilateral and caudal to the left kidney, with the supernumerary kidney being smaller than the native kidney [3]. The supernumerary kidney is thought to arise from an abnormal division of the nephrogenic cord into two separate metanephric blastemas during the fifth to seventh week of gestation [5]. This process yields two kidneys with partial or duplicated ureteral buds which may eventually lead to the formation of an accessory kidney. These can occur with two separate collecting systems or as a partially duplicated system where one ureter drains into the other. In even rarer cases, supernumerary kidneys have been reported to occur with an ectopic ureter that drains into other structures, such as the vagina [6]. These cases will present with urinary incontinence.

Although there is a very low incidence of supernumerary kidney, associated congenital abnormalities have been reported. These include horseshoe kidney malformation, ventricular septal defects, neural tube defects, and cloacal abnormalities, such as urethral atresia, vaginal atresia, ectopic ureter implantation, imperforate anus, and duplication of urethra penis and urethra [7-8].

## Conclusions

Supernumerary kidneys are extremely rare. This case is even more unique in two regards. First, the supernumerary kidney is located on the right. Second, the supernumerary kidney is fused to the lower pole of the native kidney. Our patient did not exhibit associated congenital abnormalities. However, his unexplained right lower quadrant pain could be due to his anatomic variation with the known associated symptom of pain. At the time of writing this article, the patient had not received any intervention due to the lack of acute findings.
